# Development of Predictive Models for the Response of Vestibular Schwannoma Treated with Cyberknife^®^: A Feasibility Study Based on Radiomics and Machine Learning

**DOI:** 10.3390/jpm13050808

**Published:** 2023-05-10

**Authors:** Isa Bossi Zanetti, Elena De Martin, Riccardo Pascuzzo, Natascha Claudia D’Amico, Sara Morlino, Irene Cane, Domenico Aquino, Marco Alì, Michaela Cellina, Giancarlo Beltramo, Laura Fariselli

**Affiliations:** 1 Department of Diagnostic Imaging and Stereotactic Radiosurgery, Centro Diagnostico Italiano S.p.A., Via Saint Bon 20, 20147 Milan, Italy; isa.bossizanetti@cdi.it (I.B.Z.); damiconatascha@gmail.com (N.C.D.); marco.ali@cdi.it (M.A.); giancarlo.beltramo@cdi.it (G.B.); 2 Health Department, Fondazione IRCCS Istituto Neurologico Carlo Besta, Via Celoria 11, 20133 Milan, Italy; elena.demartin@istituto-besta.it; 3 Neuroradiology Unit, Fondazione IRCCS Istituto Neurologico Carlo Besta, Via Celoria 11, 20133 Milan, Italy; domenico.aquino@istituto-besta.it; 4 Radiotherapy Unit, Neurosurgery Department, Fondazione IRCCS Istituto Neurologico Carlo Besta, Via Celoria 11, 20133 Milan, Italy; sara.morlino@istituto-besta.it (S.M.); irene.cane@istituto-besta.it (I.C.); laura.fariselli@istituto-besta.it (L.F.); 5 Bracco Imaging S.p.A., Via Caduti di Marcinelle 13, Milan 20134, Italy; 6 Radiology Department, ASST Fatebenefratelli Sacco, Piazza Principessa Clotilde 3, Milan 2021, Italy

**Keywords:** machine learning, radiomic, vestibular schwannoma, Cyberknife, personalized medicine

## Abstract

Purpose: to predict vestibular schwannoma (VS) response to radiosurgery by applying machine learning (ML) algorithms on radiomic features extracted from pre-treatment magnetic resonance (MR) images. Methods: patients with VS treated with radiosurgery in two Centers from 2004 to 2016 were retrospectively evaluated. Brain T1-weighted contrast-enhanced MR images were acquired before and at 24 and 36 months after treatment. Clinical and treatment data were collected contextually. Treatment responses were assessed considering the VS volume variation based on pre- and post-radiosurgery MR images at both time points. Tumors were semi-automatically segmented and radiomic features were extracted. Four ML algorithms (Random Forest, Support Vector Machine, Neural Network, and extreme Gradient Boosting) were trained and tested for treatment response (i.e., increased or non-increased tumor volume) using nested cross-validation. For training, feature selection was performed using the Least Absolute Shrinkage and Selection Operator, and the selected features were used as input to separately build the four ML classification algorithms. To overcome class imbalance during training, Synthetic Minority Oversampling Technique was used. Finally, trained models were tested on the corresponding held out set of patients to evaluate balanced accuracy, sensitivity, and specificity. Results: 108 patients treated with Cyberknife^®^ were retrieved; an increased tumor volume was observed at 24 months in 12 patients, and at 36 months in another group of 12 patients. The Neural Network was the best predictive algorithm for response at 24 (balanced accuracy 73% ± 18%, specificity 85% ± 12%, sensitivity 60% ± 42%) and 36 months (balanced accuracy 65% ± 12%, specificity 83% ± 9%, sensitivity 47% ± 27%). Conclusions: radiomics may predict VS response to radiosurgery avoiding long-term follow-up as well as unnecessary treatment.

## 1. Introduction

Vestibular Schwannoma (VS) is a rare benign primary tumor that develops from Schwann cells of the eighth cranial nerve and has an incidence of 1.1/100,000 inhabitants, representing 7% of all intracranial tumors [1]. It is generally a slow-growing tumor, appearing more frequently during the fifth decade and its symptoms are related to the size. The first symptoms can be hearing decrease or loss, tinnitus, face numbness, and decreased balance, while larger VS can cause more disabling symptoms such as facial paralysis or intracranial hypertension in extremely severe cases [1].

Nowadays, there are no randomized trials to guide the management of patients with VS, but the current guidelines suggest three different clinical approaches for VS [2]: observation through follow-up, surgical removal, and stereotactic radiotherapy/radiosurgery (SRS). The choice of treatment depends on the tumor size and the severity of symptoms with the primary objective to preserve the patient’s quality of life.

Surgery is usually the treatment of choice for large (more than 3 cm diameter) and symptomatic lesions. Patients with small tumors are usually monitored through scheduled magnetic resonance imaging (MRI) investigations. In this way, should the tumor grow or become symptomatic, it can be treated with surgery or SRS [3,4]. The latter can also be considered in cases where traditional surgery is not indicated, for example, in patients with neurofibromatosis, comorbidities, or elderly patients [5]. The use of SRS is increasing and nowadays is the treatment choice for small- to medium-sized VS, with favorable overall tumor control rates around 90% at five years depending on series [6,7].

SRS for VS can be performed using different systems and machines, all producing a high rate of functional sparing of the facial and trigeminal nerves [8,9]. However, complications of SRS may cause mild side effects (e.g., tinnitus, worsening of hearing or balance) or severe ones such as trigeminal neuralgia, delayed hearing loss, and facial palsy showing potential cranial nerves dysfunctions in a few patients [10,11].

Currently, it is not possible to identify patients who do not respond to treatment, and it is necessary to wait at least two years to assess whether the SRS has been effective or not, causing diagnostic uncertainty and delays in any further treatments [8,12].

This issue could be addressed and solved by applying artificial intelligence methods to the MRI images acquired during the pre-treatment phase, especially using Radiomics. 

Radiomics is one of the emerging research fields of the greatest interest in the diagnostic imaging world. Machine learning (ML) and deep learning algorithms have been used to analyze a large amount of data from images and integrated into predictive models to improve and refine the radiological diagnosis and the clinical management of prognostic property patients.

In neuro-oncology, radiomics is mainly based on the analysis of conventional MRI. Several studies have investigated the usefulness of radiomics for classification, molecular characterization, differentiation of treatment-related changes from tumor progression, and prediction of local response primarily in patients with gliomas and brain metastases [13,14,15].

Recent studies evidenced the potential of applying radiomics combined with a Gamma knife, but, to the best of our knowledge, there are no publications on predicting VS response to Cyberknife treatment using a radiomic approach [16,17,18,19].

Therefore, the purpose of this study is to examine the prognostic properties of radiomic features extracted in pre-SRS contrast-enhanced T1-weighted MRI to predict VS outcome at 24 and 36 months after SRS Cyberknife treatment.

## 2. Materials and Methods

### 2.1. Patient Cohort

In this retrospective observational multicenter study, we collected a cohort of 108 patients diagnosed with VS who underwent a Cyberknife radiosurgery in single or multiple sessions at both institutions, CDI Centro Diagnostico Italiano S.p.A. and Fondazione IRCCS Istituto Neurologico Carlo Besta (Milan, Italy) from 2004 to 2016.

We included all patients with unilateral VS of the eighth cranial nerve with a pre-treatment T1-weighted contrast-enhanced MRI and follow-up MRI exams performed both at 24 and 36 months. Patients with an incomplete examination, imaging artefacts, neurofibromatosis, bilateral lesions, and those who underwent previous brain radiotherapy were excluded. The Ethics Committee of Fondazione IRCCS Istituto Neurologico Carlo Besta approved this study (Approval date: 3 April 2019) and informed consent was obtained from all patients. Data were shared between Centers and managed following the international guidelines and GDPR regulation [20].

We collected the following baseline clinical data for each patient: age, sex, presence of symptoms, chronic diseases, 5th or 7th cranial nerve deficit, audiometric data (if present), and previous surgery. Moreover, tumor-specific aspects (intra- or extra-canalicular extent, tumor volume and diameters, and tumor laterality) and radiation parameters (total dose, number of fractions, fractioned dose, isodose, and collimators) were recorded. Audiometric data were collected to describe the hearing function which was assessed according to the guidelines of the American Academy of Otolaryngology–Head and Neck Surgery Foundation (AAO–HNS) [21].

The characteristics of the collected patients are summarized in Table 1. The mean age was 61 years, and 62/108 (57%) patients were women.

According to the volume outcome and diameters measurement, comparing the pre-treatment and the follow-up MR images, and considering both follow-up times independently, each patient was assigned to one of the following classes: “decreased”, “increased”, and “stable”. The first class named “decreased” included all patients with any reduction in lesion size. The second class named “increased” included all patients with an increase in lesion size, while the third group included all patients with volume stability. 

An increased volume was observed for 12 out of 108 patients at 24 months; 7 of those 12 patients had an increased volume also at 36 months, while the other 5 were assigned to the “stable” class; at 36 months, 5 patients showed an increased volume for the first time, therefore, a total of 12 patients had increased volume at 36 months. 

For each of the two-time points, the classes “decreased” and “stable” have been merged into a “non-increased” class to train the ML algorithms in identifying patients not responding to treatment (the “increased” class).

### 2.2. Images Acquisition

Brain images were acquired with 1.5T MRI systems at both institutes. The common MRI protocol included 3D T1-weighted MR images acquired pre- and post-contrast administration (Gadovist, Bayer 1.0 mmol/mL; Magnevist, Bayer 0.2 mmol/kg; ProHance, Bracco 0.1 mmol/kg). The sequence parameters of each scanner are reported in Table 2.

### 2.3. Radiosurgical Treatment

Radiosurgery was performed using a Cyberknife system (Accuray, Inc., Sunnyvale, CA, USA). In our cohort, two treatment schedules were applied: 57 patients (53%) were treated with a median total dose of 12 Gy in a single fraction (range 12–13 Gy) and 51 patients (47%) were treated with a median total dose of 18 Gy (range 18–21 Gy) in three fractions (median 6 Gy/fraction), according to the VS size and patient clinical status. The median isodose surface was 80% (range 75–86%). Furthermore, 7.4% of patients were treated with a multi-leaf collimator, while the others with a fixed collimator with sizes going from 5 mm to 20 mm.

### 2.4. Segmentation

Semi-automatic segmentation of the tumor was performed by an expert Radiation Oncologist on the post-contrast T1-weighted image using 3D Slicer image analysis software (https://www.slicer.org/, accessed on 9 May 2023) [22]. By performing this segmentation process for each image layer (slice), the lesion volume of interest (VOI) was obtained.

### 2.5. Image Pre-Processing

We performed multiple steps of image pre-processing, to use the images for the subsequent radiomic feature extraction. First, all the post-contrast T1-weighted 3D images were resampled to have isotropic voxels of dimensions of 1 × 1 × 1 mm^3^. Second, the T1-weighted 3D images were processed to correct low-frequency intensity non-uniformity (also known as bias field) using N4BiasFieldCorrection, a tool belonging to the ANTs package (http://stnava.github.io/ANTs/ accessed on 9 May 2023). Bias fields are due to inhomogeneity in the magnetic fields of MRI machines. Visually, the image appears blurred, with less evident edges, and with altered intensity values of image pixels, so that the same tissue appears with different gray level distributions across the image. Though low variation does not impact clinical diagnosis, it can degrade the performance of image-processing algorithms that are based on the spatial invariance of the processed image. Thus, it is common practice to perform a correction of this bias before proceeding with the analysis [23]. Third, the brain image was segmented into 3 tissue classes (Gray matter, White matter, CSF) by the unified segmentation tool integrated into the Statistical Parametric Mapping v12 tool (SPM12) (http://www.fil.ion.ucl.ac.uk/spm/ accessed on 9 May 2023) for deskulling [24]. The brain mask was obtained as the union of the three tissue masks and applied to the corrected T1 volumes to estimate the mean value and standard deviation of the brain parenchyma. Finally, to standardize voxel intensities across multiple MRI scanners, images were z-transformed (i.e., the brain mean value was subtracted from each voxel value, and then this difference was divided by the brain standard deviation) z-score maps were obtained.

### 2.6. Features Extraction

Radiomic features of the volume of interest were extracted from z-score maps of T1-weighted images using the open-source Python package “Pyradiomics” [25]. Gray value discretization of the maps was performed using fixed bin counts equal to 32. Next, the following classes of features were extracted: shape (*n* = 14), first-order (*n* = 18), gray level co-occurrence matrix (GLCM, *n* = 24), gray level dependence matrix (GLDM, *n* = 14), gray level run length matrix (GLRLM, *n* = 16), gray level size zone matrix (GLSZM, *n* = 16), and neighboring gray tone difference matrix (NGTDM, *n* = 5). More details on these features are available online at the Pyradiomics website (https://pyradiomics.readthedocs.io/en/latest/features.html accessed on 9 May 2023). All features except those of the shape class were also extracted from wavelet-transformed z-score maps, based on the 8 possible combinations of applying either a high or a low pass filter in each of the three dimensions of the image.

### 2.7. Feature Selection, Model Development and Test

The whole dataset was split into a development set and a test set according to a 5-fold cross-validation scheme (outer loop), stratifying the folds to have similar outcome distribution. For each data split, three rounds of analysis were performed: (1) data cleaning, (2) feature selection, and (3) training four ML algorithms to predict an increase in the lesion at 24 or at 36 months after radiosurgery. Of note, in the second and the third rounds of analysis, a further split of the development set was performed according to a 3-fold cross-validation scheme (inner loop); this procedure is also called nested cross validation. Details on the three rounds of analysis are reported below. 

In the first round, missing clinical data were inputted using bootstrap aggregating (bagging) regression trees; the trees were trained on the development set and then used to predict missing data in the test set to avoid any data leakage. No radiomic feature was missing for any patient. Next, radiomic features acquired from each Centre were standardized to account for possible differences due to the use of multiple MRI scanners. Standardization of each radiomic feature was performed in the development set by subtracting the mean value and dividing by the standard deviation. Then, using the means and the standard deviations computed from that set, radiomic features were standardized also in the test set. 

The second round of analysis was conducted only on the development set and entailed a feature selection step performed through a logistic regression model with the Least Absolute Shrinkage and Selection Operator (LR-LASSO). This model allowed the removal of irrelevant or redundant features before the classifier training. To fit the LR-LASSO model, the development set was further split into the training and validation sets according to a 3-fold cross-validation scheme (inner loop). For each data split, the two classes (“increased” or “not increased”) were first balanced in the training set using a synthetic minority over-sampling technique (SMOTE) [26,27]. This allowed for augmenting the training set with plausible synthetic examples that were relatively close in feature space to existing examples from the minority class (“increased”). The LR-LASSO model was then built on the training set changing the parameter lambda over a set of 100 possible values in the range 0.001–0.300. Each lambda value led to a different set of selected features; the corresponding fitted LR-LASSO model was used to predict the class of the patients in the validation set. The lambda value (and consequently the set of features) that yielded the highest balanced accuracy (i.e., average between sensitivity and specificity) on the validation set was selected.

In the third round of analysis, the following four ML algorithms were chosen: Random Forest (RF), Neural Network (NNet), Support Vector Machine (SVM) with radial kernel, and eXtreme Gradient Boosting (XGBoost). These algorithms were fitted on the features selected at the previous step by LR-LASSO on the development set. Hyperparameter tuning was performed on another partition of the development set into the training and validation set, based on a 3-fold cross-validation procedure (inner loop). In a similar way to the second round of analysis, for each internal split of the development set, a SMOTE procedure was used to balance the classes on the training set. Next, the 4 algorithms were fitted on the training set based on different sets of hyperparameters, and the best hyperparameters for each algorithm were selected based on the highest balanced accuracy that was obtained on the corresponding validation set. The fine-tuned models were finally evaluated on the test sets. The final classification metrics (balanced accuracy, sensitivity, and specificity) of the models were computed as the average (±standard deviation) metrics across the 5 test sets of the outer loop. Of note, a different set of features was used for each test set, because it was generated by a different development set. We recorded the frequency in which each feature appeared across the 5 runs of (external) cross validation to evaluate the features that contributed the most to the classification. All statistical analyses were performed in R (version 4.2.1) with the “caret” package.

## 3. Results

**Figure 1:**
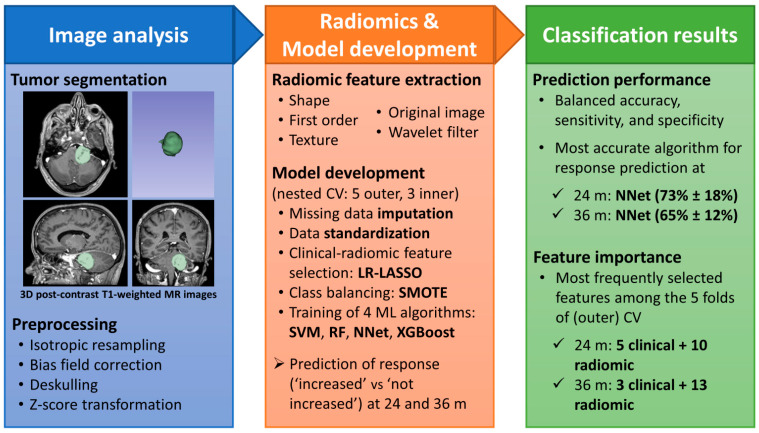
Analysis pipeline and summary of results. Abbreviations: MR = magnetic resonance; CV = cross-validation; LR-LASSO = logistic regression with least absolute shrinkage and selection operator; SMOTE = synthetic minority oversampling technique; SVM = support vector machine with radial kernel; RF = random forest; NNet = neural network; XGBoost = extreme gradient boosting.

A total of 851 radiomic features were extracted for each of the 108 patients included in the present study. Increased tumor volumes concerning the pre-treatment evaluation were observed in a group of 12 patients at 24 months after treatment and in a group of 12 patients (seven already present at 24 months and five with a novel increase) at 36 months, as described in the “Patient Cohort” section. Figure 1 recapitulates the analysis pipeline and shows a brief summary of the main results obtained in the present study.

The top clinical and radiomic features selected more than once to predict the response at each time point and their frequency over the five runs of (external) cross validation are reported in Table 3. When analyzing images obtained at 24 months after treatment, the most selected features were five clinical (age, tumor laterality, total dose, intra-, or extra-canalicular disease, fifth or seventh cranial nerve deficit) and ten radiomic features (six texture and four first-order features). As for the 36 months response prediction, age, fifth or seventh cranial nerve deficit and four of the radiomic features selected at twenty-four months (“Zone Entropy”, “Energy”, “MCC”, and “Small Area High Gray Level Emphasis”) were frequently chosen. Moreover, isodose, the shape feature “Flatness”, three textures, and four first-order features were also selected.

Classification results of the four ML algorithms on the test set are reported in Table 4 for the prediction of the increase in tumor size at 24 months and 36 months. The Neural Network was the best ML algorithm for predicting the response at 24 months (average test set balanced accuracy 73%, sensitivity 60%, and specificity 85%) and 36 months (balanced accuracy 65%, sensitivity 47%, and specificity 83%).

## 4. Discussion

In this work, quantitative radiomic analysis on pre-treatment MR images and ML algorithms were combined to predict tumor volume increase at two different time points (24 and 36 months after SRS) in patients affected by VS treated with Cyberknife. Classification results with NNet were satisfactory for the prediction at 24 months (73% balanced accuracy, with 60% sensitivity and 85% specificity), while they decreased at 36 months (65% balanced accuracy, with 47% sensitivity and 83% specificity).

To the best of our knowledge, there are no previous studies on predicting VS response after Cyberknife treatment using a radiomic approach. Therefore, we compared our methods and results with similar works where patients underwent Gammaknife treatment. The obtained balanced accuracy of 73% is comparable with the result of Langenhuizen et al. [16] who obtained an accuracy of 70% in distinguishing patients with volumetric response from patients without analyzing 85 patients treated for unilateral VS.

The developed methods and results obtained in our work were also compared with those described by Lee et al. [17] who included 330 patients and distinguished between responders and non-responders. After using a LASSO followed by an SVM model, they obtained higher accuracy values (AUC = 0.91) than ours for distinguishing tumor response from non-response at 24 months after treatment, likely due to the larger number of patients included in their study. 

Our results showed that 72% and 65% of the patients were correctly classified at the two time points, 24 and 36 months, respectively, with a specificity of 85% and 83%. However, the corresponding sensitivities were lower (60% and 47%, respectively), probably due to the small “increased” population which contains only 5% of the dataset. 

Given the size of the available dataset and the high imbalanced distribution, particular attention was given to increase the sample size during training using SMOTE as a minority class oversampling technique and to develop the algorithms using nested cross-validation, so that classification results could be reliably estimated, and their generalizability could be fairly assessed. 

As for the most relevant features selected by LASSO, a set of common features were selected across the five external folds and for the two target points, such as the age at the time of treatment, the fifth or seventh cranial nerves deficit, and both first order and texture features. Interestingly, among the radiomic features, the maximal correlation coefficient (MCC, a GLCM feature), related to the complexity of the texture, and the “Small Area High Gray Level Emphasis”, which measures the proportion in the image of the joint distribution of smaller size zones with higher gray-level values (i.e., areas of the tumors that are more enhanced by the contrast) were selected in most of the combinations.

Traditional Machine Learning models, such as Random Forest and Support Vector Machine, proved insufficient to model the complexity of the data. The Neural Network, having a more complex architecture, demonstrated greater potential in learning how to discriminate patients with increased tumor volume from the others. 

Although the achieved results are promising, further research is needed for several reasons.

One of the main limits of this study is the restricted dataset and the class with increasing tumor volume that represented only 5% of the entire cases. A SMOTE algorithm has been used to produce synthetic samples from the minority class, estimating both clinical and radiomic features. The literature also reports promising methods for the synthesis of MR images [28]. Further research should be performed to assess if such methods can further improve models’ performance and provide synthetized data with more realistic radiomic features in terms of distribution and variability.

Furthermore, expanding the training dataset would allow the testing of more complex architectures, such as end-to-end Deep Learning frameworks. Being very data hungry, such approaches had to be excluded from the current work, but they could be investigated to assess if treatment outcomes could be predicted directly from the MR image without explicit feature extraction.

Another confounder is the retrospective design. The first patients were treated in 2004: MRI intensities can vary between subjects and protocols have improved over time. Despite our attempt to minimize the impact of these variations, they may still be present albeit at a reduced level. It is, therefore, possible that the currently used conventional MRIs exhibit more detailed radiomic features, leading to improved SRS outcome prediction. 

Furthermore, we analyzed only T1-weighted images with contrast enhancement; Yang et al. [18] achieved an accuracy of 88.4% in the prediction of long-term outcomes after radiosurgery based on five radiomic features describing, in addition to inhomogeneity of contrast enhancement, the variation of T2-weighted intensity. Due to the information contained in T2-weighted images, in a future prospective study, information from other MRI sequences could be added and analyzed, since they were not included in our treatment planning protocol. 

Moreover, Langenhuizen et al. [16] noted that the most predictive features and the most reliable results in prediction were obtained in tumors larger than 5 cm^3^. Since in our dataset, the volume ranged broadly from 0.15 to 16 cm^3^, we believe further analysis would benefit from volume size stratification. 

It would also be important from a clinical point of view to increase the number of patients and collect MRI data at other time points to improve the robustness of the approach and to evaluate both the possible role of the pseudoprogression and late response to treatment. Moreover, a delta-radiomic approach (i.e., the evaluation of changes in radiomic features over time) could also be tested, as conducted by Zhang and colleagues to distinguish radiation necrosis from tumor progression in brain metastases after radiosurgery [29].

## 5. Conclusions

The current work shows the feasibility of a radiomic approach to discriminate patients with tumor volume increase from patients without after a Cyberknife^®^ treatment on an individual basis by analyzing routinely collected MRI images. From these results, further research could be conducted to create a clinical decision support system for physicians and patients which would facilitate the management of vestibular schwannoma. To this end, the inclusion of untreated cancer cases in the analysis pipeline can also result in a benefit.

## Figures and Tables

**Table 1:** Characteristics of 108 patients included in the study.

Variable		Value (No. or Mean)
Age, mean (range)		61 years (34–83)
Sex	Female	62
	Male	46
Symptoms		77
Chronic diseases		41
5th or 7th cranial nerve deficit		16
Audiometric data	A	1
	B	13
	C	45
	D	20
Previous surgery		19
Intra- or extra-canalicular disease	Intra	17
	Extra	13
	Intra and Extra	78
Tumor volume, mean (range) mm^3^		3108 (154–16,239)
Tumor laterality	Left	52
	Right	56

**Table 2:** Sequence parameters used by the different MR scanners.

Characteristics	Magnetic Resonance System		
	CDI Centro Diagnostico Italiano		IRCCS Istituto Carlo Besta
	GE Signa Excite 1.5 T	Philips Achieva 1.5 T	Philips Achieva 1.5 T
Pulse sequence	T1-w 3D	ST1w-3D-Iso sense	T1 3D TFE mdc
TR [ms]	15.27	25	7.16
TE [ms]	6.93	4.5	3.21
Slice thickness [mm]	1	1	1
Slice spacing	0	0	0
Pixel spacing [mm/mm]	0.97/0.97	1/1	1/1

**Table 3:** Top selected predictive features.

Feature Type	Most Frequently Selected Features for Predicting Response to Treatment:			
	at 24 Months		at 36 Months	
	Name	Frequency	Name	Frequency
Clinical	Age Laterality right Laterality left Total dose Deficit 5–7th Extra-intra canalicular	5 5 4 2 2 2	Isodose Deficit 5–7th Age	3 3 2
Radiomic Shape	-	-	Flatness	3
Radiomic First order	HHL-Median HHH-Median LHL-Minimum LLH-Energy	4 2 2 2	LHL-Skewness HHL-Maximum LLH-Energy HLH-Kurtosis LLH-90Percentile	4 3 2 2 2
Radiomic Texture	HHL-GLSZM- SmallAreaHighGrayLevel-Emphasis HLH-GLSZM- HighGrayLevelZoneEmphasis HHL-GLSZM- SmallAreaLowGrayLevel-Emphasis HHH-GLSZM-ZoneEntropy Original-GLCM-MCC HHL-GLCM-MCC	4 3 2 2 2 2	HHH-GLSZM-ZoneEntropy HLH-GLRLM-RunEntropy HHL-GLCM-ClusterShade HHL-GLRLM-ShortRunHigh- GrayLevelEmphasis HHL-GLSZM-SmallAreaHigh- GrayLevelEmphasis HHH-GLSZM-SmallArea-Emphasis HHH-GLCM-MCC	3 3 3 2 2 2 2

The top selected features for predicting the response to treatment at 24 and 36 months after radiosurgery. The number of times a feature was selected among the folds of the cross-validation procedure is also reported (maximum is 5). Radiomic features were extracted from the original processed images and the processed images after wavelet transformations (8 possible combinations of high [H] and low [L] pass filters along the 3 dimensions). The description of each radiomic feature is available at https://pyradiomics.readthedocs.io/en/latest/features.html# accessed on 9 May 2023.

**Table 4:** Summary of classification performance.

Time Point	Classification Metric	Machine Learning Algorithms			
		SVM	RF	NNet	XGBoost
24 months	Balanced Accuracy %	56.5 ± 15.9	55.3 ± 14.1	**72.6** **± 17.7**	57.4 ± 22.2
	Sensitivity %	20.0 ± 27.4	20.0 ± 27.4	60.0 ± 41.8	30.0 ± 44.7
	Specificity %	92.9 ± 7.7	90.6 ± 3.2	84.7 ± 12.2	84.7 ± 12.2
36 months	Balanced Accuracy %	52.4 ± 13.2	56.9 ± 17.8	**64.9** **± 11.8**	51.7 ± 10.5
	Sensitivity %	13.3 ± 18.3	20.0 ± 29.8	46.7 ± 27.4	16.7 ± 23.6
	Specificity %	91.5 ± 09.2	93.9 ± 8.7	83.2 ± 9.4	86.7 ± 13.0

Performance of our four ML algorithms on the held-out test set, for predicting the response to treatment at 24 and 36 months after radiosurgery. Numbers are Mean ± Standard Deviation across 5 folds of the outer loop. Numbers in bold indicate the ML algorithm with the highest balanced accuracy at the two time points. ML = machine learning; SVM = Support Vector Machine with radial kernel; RF = Random Forest; NNet = Neural Network; XGBoost = eXtreme Gradient Boosting.

## Data Availability

Data available on request to the corresponding authors.
